# A Behavioral Assay for Investigating the Role of Spatial Memory During Instinctive Defense in Mice

**DOI:** 10.3791/56988

**Published:** 2018-07-21

**Authors:** Ruben Vale, Dominic Evans, Tiago Branco

**Affiliations:** ^1^UCL Sainsbury Wellcome Centre for Neural Circuits and Behaviour; ^2^MRC Laboratory of Molecular Biology

**Keywords:** Behavior, Issue 137, Spatial memory, spatial learning, navigation, path integration, innate, flight, freezing, looming stimulus, ultrasound, shelter

## Abstract

Evolution has selected a repertoire of defensive behaviors that are essential for survival across all animal species. These behaviors are often stereotyped actions elicited in response to innately aversive sensory stimuli, but their success requires enough flexibility for adapting to different spatial environments, which can change rapidly. Here, we describe a behavioral assay to evaluate the influence of learned spatial knowledge on defensive behaviors in mice. We have adapted the widely used Barnes maze spatial memory assay to investigate how mice navigate to a shelter during escape responses to innately aversive sensory stimuli in a novel environment, and how they adapt to acute changes in the environment. This new assay is an ethological paradigm that does not require training and exploits the natural exploration patterns and navigation strategies in mice. We propose that the set of protocols described here are a powerful means of studying goal-directed behaviors and stimulus-triggered navigation, which should be of interest to both the fields of instinctive behaviors and spatial memory.

**Figure:**
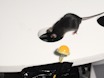


## Introduction

Instinctive defensive behaviors are largely considered to be hardwired stimulus-responses, such as the C-start movement in fish and amphibians, which moves the animal away from a threat source^1^. However, defensive behaviors can be more adaptive if they flexibly take into account information learned about the current environment. One example of such flexibility is the switch from escape to freezing shown by rodents when faced with threat, depending on prior knowledge about the presence or absence of a shelter in the environment^2^,^3^. Other examples of flexibility in innate behaviors include adapting the flight initiation threshold or escape speed depending on the distance between a prey and its shelter^4^, distance to threat^5^,^6^, and previous experience^11^,^12^, as well selecting different defensive strategies depending on the sensory properties of the aversive stimulus^7^, or even suppressing defensive behavior in face of competing motivations such as hunger^8^,^9^,^10^. The dependency of action selection on acquired knowledge about the spatial features of the environment makes instinctive defensive behaviors in mice a powerful model for studying goal selection, spatial memory and navigation. Here, we describe the adaptation of a commonly used behavioral task, the Barnes maze (BM) spatial memory assay^13^, to determine the influence of the spatial environment on defensive action selection in mice, and their navigation strategies when escaping towards a shelter.

The standard Barnes maze used to study learning and spatial memory in mice consists of a circular ~90 cm diameter platform with 20 equally spaced holes, of which 19 are closed, and one leads to an underground shelter which mice seek in order to avoid the open-field environment of the platform. Often, weak aversive stimuli (buzzer, bright light, fan) are used continuously throughout the assay to make the environment aversive and thus promote entry into the shelter^14^. In the most commonly used assay^15^,^16^, the animal has one habituation trial where it is guided to the shelter manually by the experimenter immediately after being positioned on the platform. This is followed by a 4-day acquisition period, where each day the mouse is allowed to navigate freely in the maze for 3 min, after which it is again manually guided to the shelter if it does not reach it during the exploration period. The last stage of the assay is one probe trial on the 5^th^ day (although a long-term seven-day memory trial is also commonly performed) when the animal explores the maze with all holes closed. Learning and long-term memory are quantified by the time taken to find the shelter and pokes in the wrong holes during the acquisition period, and by the time spent near the closed shelter hole in the probe trial. Typical results show a decrease in the number of errors and latency to reach the shelter during acquisition, and an above-chance proportion of time spent in the quadrant containing the closed target hole in the probe trial^15^.

While several variants of the BM assay have been previously described^17^,^18^,^19^, the paradigm we describe here has three fundamental changes from the standard assay. First, the animal is left to explore the maze and find the shelter on its own, and testing is done in the same session, shortly after the shelter has been found, during periods where the animal is engaged in exploratory behavior. Although this setting does not test long-term memory of the shelter location, it is designed to be a naturalistic scenario that mimics the exploration of novel territory, under the threat of predation. In addition, it allows testing of how animals adapt to acute changes in the environment, such as sudden changes in landscape. Second, a key aspect of our assay is that the experimenter never forces the animal in or out of the shelter, which can disorient the animal and exclude path integration^20^ as a viable navigation strategy^21^. Path integration is a navigation strategy that uses self-motion cues, such as proprioceptive and vestibular cues, arising from the integration of motor outflow, to update the current position of the animal and navigate towards a goal, which is not possible if the animal is passively moved by the experimenter. Third, we use innately aversive visual^22^ and auditory^23^ stimuli to elicit escape to shelter, which is easily distinguished from ongoing foraging behavior and allows the assessment and quantification of specific navigation strategies used during defense from imminent threats. We propose that this assay will be useful for dissecting the role of spatial memory in the selection and implementation of defensive behaviors, and more generally to the broader study of goal-directed navigation and short-term spatial memory. The protocols described here were introduced by Vale *et al*. in 2017, to which we refer the readers for more in depth discussion of the rationale for experiments and results.

## Protocol

All experiments were performed under the UK Animals (Scientific Procedures) Act of 1986 (PPL 70/7652) following local ethical approval.

### 1. Setting up the Behavioral Apparatus

NOTE: The main component of the behavioral apparatus is a variant of the Barnes maze^13^ consisting of a white acrylic circular platform (92 cm in diameter) with 20 equidistant, 5 cm diameter, circular holes, located radially, 5 cm from the edge of the platform. 19 of these are to be closed with black plastic plug (5 mm deep), while the remaining hole leads to a black Perspex shelter (dimensions 15 x 5.8 x 4.7 cm) that mice can easily enter and exit. The central area of the arena consists of a fixed circular platform (22 cm in diameter) and the periphery is mounted on a frame that allows rotation over 360 degrees through a computer-controlled stepper motor. The platform is elevated 45 cm from the floor to prevent the mice from climbing down. See schematic drawing in Figure 1.

1. Attach visual cues to the periphery of the platform (2D or 3D symbols with different shapes, colors and patterns), at least 2 cm away from the edge to prevent mice from reaching them. NOTE: Examples of visual cues are plain blue squares of 262 cm^2^, or circles (diameter of 15.5 cm) filled with diagonal purple stripes.
2. Add ~2 g of bedding from the mouse's home-cage to the inside of the shelter to serve as a local olfactory cue. Optionally, position a light emitting diode (LED) at the entrance of the shelter to provide a local visual landmark cue.
3. To isolate the mouse from external, distal visual cues, assemble an octagonal or cylindrical wall, extending 40 cm above and 15 cm under the platform surface, around the arena, positioned 10 cm away from the edge of the platform.
4. Position an ultrasound speaker 50 cm above the center of the platform, to deliver aversive auditory stimuli.
5. Place a translucent screen 64 cm above the arena to back-project overhead aversive visual stimuli using a projector.
6. The light provided by the projector will illuminate the behavioral apparatus; calibrate it so that there is 5-10 lx at the center of the platform surface. Place infrared lights and an infrared camera above the platform, with a long-pass filter (>700 nm) to prevent projector flicker in the acquired video.
7. Track the position of the mouse online, using either commercially available software or a suitable algorithm. For tracking black mice on a white arena, a simple thresholding operation followed by calculation of the mask centroid is appropriate.
8. Ideally, place the behavioral apparatus inside a sound- and light-proof box, to prevent external cues from affecting the experiment.

### 2. Delivering Innately Aversive Stimuli

NOTE: The stimuli described below can be generated with a wide range of different software packages, including LabVIEW and Matlab.

1. Visual stimulus
  1. Project the stimuli on to the screen positioned 64 cm above the arena. The stimulus consists of a linearly expanding dark circle on a grey background^22^ (Weber contrast = -0.98, luminance = 7.95 cd/m^2^). NOTE: The standard circle should ideally be centered directly above the position of the mouse, subtending a visual angle of 2.6° at onset and expands linearly at 224°/s over 200 ms to 47.4°, at which it remains for 250 ms before offset.
  2. When delivering the visual stimulus, center it above the mouse. This can be achieved either by waiting until the mouse passes through a pre-defined region in the arena, or by tracking the mouse position online and using the tracking coordinates to define the center of the circle. NOTE: Although we suggest projecting the visual stimulus directly above the mouse, defensive behaviors can also be elicited if the same stimulus is projected ahead of the mouse (while still parallel to the floor). In our experience, this is the case at least until 30 cm ahead of the mouse's position.
2. Auditory stimulus
  1. Trigger a stimulus consisting of a train of three frequency-modulated up-sweeps from 17 to 20 kHz^23^ over 3 s, lasting 9 s in total. The sound pressure level (SPL), measured at the platform level, should be in a range between 73 and 78 dB.
  2. Connect a soundcard and an amplifier to the ultrasound speaker positioned above the arena to deliver the auditory stimulus.

### 3. Animals:

1. Use male or female C57BL/6J mice, between 6 and 24 weeks old. Maintain the animals on a 12 h light cycle with free access to chow and water.
2. Ensure that the mice are single housed at least 72 h before the behavioral assay and test them during the light-phase of the light cycle. NOTE: We find that the baseline exploratory behavior of single-housed mice is more comparable across animals than across group-housed mice. In addition, animals with surgical implants for neural activity recording or manipulation often need to be single housed, and it can thus be beneficial to have control datasets in the same housing conditions.

### 4. Standard Behavioral Assay:

1. Meticulously clean the surface of the platform with 70% ethanol or acetic acid to remove unwanted olfactory cues. Rinse the shelter with water and wash it with 70% ethanol, after which it should be rinsed with water again to lessen the ethanol odor.
2. Dry both the platform and the shelter thoroughly, as the mouse may avoid entering the shelter if it is wet.
3. Randomize the location of the shelter in the platform for each trial by rotating the platform before the experiment. The shelter location will be anchored in the arena but will be rotated in relation to the behavioral apparatus enclosure.
4. Bring the mouse to the experiment room in its home-cage and place the cage on top of the testing platform for a 10 min acclimatization period.
5. Remove the mouse from its home-cage and avoid retrieving by the tail: either cup it or let it climb onto an enrichment item that can be lifted. Picking mice by the tail has been show to increase anxiety levels and thus may affect behavioral responses to threat^24^.
6. Gently place the mouse in the center of the arena and start the video-acquisition.
7. Allow for a 7 min habituation period, during which no aversive stimulus is presented. Ensure that the mouse has enough time to find and enter the shelter at least once. Consider it as an entry if all four limbs are inside the shelter. 7 min should be enough for the majority of the mice to find and enter the shelter, but in case this does not happen, extend the habituation period by additional periods of 5 min until at least one entrance has been verified.
8. Trigger the delivery of the aversive stimulus (auditory or visual) either manually by observing the live video feed, or automatically by tracking the position of the animal online and pre-defining a region of interest in the arena for triggering stimulus-delivery once the animal reaches it. The visual stimulus is usually delivered directly above the animal using the online video tracking coordinates.
9. To elicit more than one escape response in the same session, let the mouse voluntarily leave the shelter. If the mouse did not successfully escape to the shelter upon the last aversive stimulus, wait at least 60 s before presenting another stimulus.
10. After the assay is finished, return the animal to its home-cage. In case further testing of the same mouse is required, wait at least 48 h before retesting.
11. Clean the platform and the shelter as described in step 4.1 and 4.2 before testing the next animal.

NOTE: Steps 5, 6 and 7 are independent variations of the standard behavioral assay and each can be performed individually.

### 5. 'Navigation Strategy' Behavioral Assay:

NOTE: The goal of this assay is to determine what cues mice use to guide escape behavior. In this assay, the movable outer portion of the platform is rotated while the mouse is on the fixed arena center, which radially displaces the shelter, the olfactory cues located inside the shelter and the proximal visual cues attached to the platform. If the mouse is following any of the cues that have been rotated in order to navigate to the shelter, it will correctly escape to the new location of the shelter, but if it uses any other cues that have remained in place, it will navigate to the previous shelter location.

1. Perform steps 4.1 to 4.8 of the standard behavioral assay described above, eliciting at least one flight response to be used as baseline.
2. Let the mouse actively leave the shelter and wait for it to navigate to the central part of the platform. Position a small dish with bedding in the center to encourage exploration and keep the animal in the central platform long enough to allow the rotation of the outer portion of the platform. Add the dish and bedding while the mouse is inside the shelter and after the successful escape that precedes the rotation of the platform. If the mouse fails to enter the shelter during the escape response, wait for it to voluntarily enter the shelter and then add the dish and bedding.
3. Use the stepper motor to rotate the platform at least 36 degrees (2 holes) and then immediately use a sensory stimulus to elicit an escape response. It is possible that the rotation triggers a startle and even initiates an escape response. NOTE: From our experience, ~55% of the mice will run outside of the central (non-rotating) platform while the external portion is still rotating, and these trials should be excluded from the analysis.
4. Clean the platform and shelter, and return the mouse to its home-cage as described in step 4.10 and 4.11.

### 6. 'Dynamic Environment' Behavioral Assay:

NOTE: This assay is designed to evaluate how mice adapt their defensive behaviors to sudden changes in the environment. In these experiments, after eliciting at least one escape response, the location of the shelter is changed and the mouse is allowed to visit it once before a subsequent escape response is triggered. If the mouse perfectly updates the new location of the shelter and computes the absence of the shelter in its previous location, it should escape to the new location. Otherwise, either it has not memorized the new location of the shelter or it has memorized both locations but prefers to escape to the old one.

1. Perform this assay after steps 4.1 to 4.8 described above, eliciting at least one flight response as a control.
2. Once the mouse voluntarily leaves the shelter, remove the shelter and re-position it in a new hole at the opposite end of the platform.
3. Allow the mouse to explore the environment until it enters the shelter at its new location.
4. When the mouse voluntarily comes out of the shelter, present it with an aversive stimulus and observe its response.
5. Use the auditory stimulus for this experiment because of its longer duration, as under prolonged threat, animals may perform additional escape responses to other locations if they fail to find the shelter at first.
6. Clean the platform and shelter and return the mouse to its home-cage as described in steps 4.10 and 4.11.

### 7. 'No Shelter' Behavioral Assay:

NOTE: The aim of this assay is to understand how the absence of a shelter in the environment affects the expression of defensive behaviors.

1. Plug all 20 holes. In this experiment, there is no shelter available in the arena.
2. After cleaning and drying the platform (steps 4.1 and 4.2), carry out steps 4.4-4.6.
3. Allow a 7 min habituation period.
4. Present the visual stimulus and observe the defensive response. The lack of a shelter promotes freezing behavior, usually lasting for the duration of the visual stimulus. Use a slowly expanding spot (*e.g*. a circle that subtends a visual angle of 2.6° at onset and expands linearly at 11.2°/s over 4 s to 47.4°, at which it remains for 1 s before offset) to facilitate the observation of freezing responses.
5. If more than one response per mouse is required, use an inter-stimulus interval of at least 60 s.
6. After obtaining the required number of responses in the absence of the shelter, introduce the shelter under one of the holes and let the mouse explore the environment for at least 5 min. 5 min will be enough time for most mice to find and enter the newly introduced shelter, but in case this does not happen, extend this period by additional 5 min blocks until at least one entry is verified.
7. Repeat steps 4.8 and 4.9, and observe escape responses performed in the presence of a shelter in the environment.
8. Clean the platform and shelter and return the mouse to its home-cage as described in steps 4.10 and 4.11.

### 8. Data Analysis:

NOTE: Each of the following data analysis steps can be performed independently.

1. Escape responses can be easily identified as a sudden acceleration following the presentation of an aversive stimulus. Quantify the latency to escape as the time between stimulus presentation and either the beginning of a head rotation movement directed at the target of the escape or the beginning of the acceleration movement, whichever comes first.
2. Freezing responses can be characterized by total immobility of the animal except for breathing movements^25^, lasting at least 0.5 s^7^. Measure the latency to freeze as the time between the presentation of the aversive stimulus and the beginning of the immobility period.
3. For each escape response, quantify the linearity of the flight path by calculating the ratio of the distance between the mouse and the shelter and the actual displacement during the flight.
4. For each escape response, calculate the accuracy by measuring the distance between the hole where the animal directed its flight to and the hole leading to the shelter. Since there are 20 holes, each hole off target represents a decrement of 10% in accuracy (*e.g*., 2 holes off target can be expressed as 80% accuracy)
5. For the 'navigation strategy' behavioral assay, quantify the accuracy of the response relative to the shelter location before the platform rotation. An average accuracy that is not significantly different from the pre-rotation accuracy indicates that the animal is not using any of the sensory cues that were rotated in order to navigate to the escape target.
6. For the 'dynamic environment' behavioral assay, classify each escape response elicited as being directed towards the new or the old shelter location, or to neither of them. Evaluate how the pattern evolves along the iterations of the elicited defensive behaviors.
7. For the 'no shelter' behavioral assay, compare the mouse's speed following the presentation of aversive visual stimulus in the presence and absence of a shelter and calculate the latency to the initiation of the defensive response. Additionally, calculate the probability of triggering a freezing response in response to the stimulus for each situation.

## Representative Results

Mice exposed to auditory or visual stimuli initiated fast escape responses with short latencies between the onset of stimulation and initiation of flight. The mean latency to escape from the visual stimulus was 202 ± 16 ms (n = 51 responses from 26 animals) and significantly longer for auditory stimulation: 510 ± 61 ms (n = 36 responses from 15 animals, *p*<0.0001 *t-test* between visual and auditory stimulation; Figure 2A) (step 8.1). Escape responses were accurately directed to the shelter (mean accuracy for visual stimulus: 97.2 ± 1.4% and 95.0 ± 1.4% for auditory stimulus, not significantly different between the two types of stimuli, *p* = 0.1655 *t-test*; Figure 2B) (step 8.4), and flight trajectories were close to a straight line (mean ratio of displacement to distance to shelter for visual stimulus 107.3 ± 1.3% and 113.9 ± 1.5% for auditory stimulus; Figure 2C) (step 8.3). Observing a short latency from the stimulus is important to distinguish stimulus-evoked behavior from homing runs, and the high linearity of the flight trajectory demonstrates that the escape response has the goal of reaching the shelter, and not simply moving away from the stimulus.

Stimulus presentation after the rotation of the outside part of the maze while the mouse was stationary in the center platform (range = 36°-90°, mean 56°) successfully elicited escape responses (step 5). All mice fled towards the previous location of the shelter, with no decrease in accuracy or linearity of flight trajectory in comparison to the trials before rotation. The mean accuracy, measured in reference to the original shelter position, was 96.3 ± 1.3% before rotation (n = 25 responses from 8 mice) and even slightly higher after rotation (100 ± 0%, *p* = 0.009 *t-test* between pre and post rotation). The mean linearity in relation to the original shelter location was 109.4 ± 5.0% pre-rotation (25 responses from 8 mice) and 109.1 ± 2.1%, post-rotation (n = 8 responses from 8 mice), *p *= 0.957 *t-test* between pre and post-rotation, Figure 3A** and B**). These results demonstrate that proximal visual cues are not used to guide the flight to the shelter (steps 8.3-8.5).

Changing the location of the shelter after eliciting one successful escape response (step 6) resulted in mice visiting the new shelter location shortly after the change (mean time to visit the new shelter = 33.1 s, range = 4-82 s). Subsequent sequential trials of sound stimulation produced a gradual increase in the probability of escaping to the new location (44.4% on the first trial), reaching 100% across all animals by the fifth trial (n = 9 animals, Figure 3C). These experiments probe the dynamics of updating the shelter location memory to adapt to changes in the environment (step 8.6).

After a 7 min acclimatization period to the maze with no shelter (step 7), the presentation of a 5 s long visual stimulus produced freezing behavior instead of escape (freezing probability = 95.2%, mean freezing time = 7.9 ± 2.7 s, n =7 animals). Subsequent introduction of a shelter in the same session and the presentation of the long visual stimulus elicited escape responses reliably, showing that mice can flexibly switch defensive strategies depending on the presence of refuge (Figure 3D) (steps 8.1 and 8.7).

**Figure:**
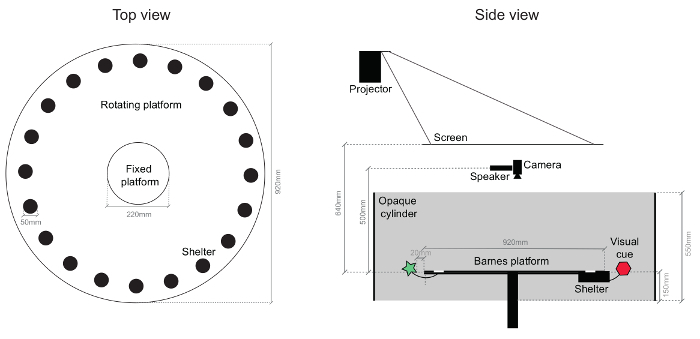

**Figure 1: Schematic of the behavioral arena.** Drawing of the main components of the behavioral arena viewed from the top (right) and from the side (left). A large fraction of the platform can be rotated, including the shelter and the attached visual cues. Dark circles in the top view are the holes to which the shelter can be attached to, which are all identical (an example shelter location is indicated). The entire arena is enclosed in a sound-dampening cabinet. Please click here to view a larger version of this figure.

**Figure:**
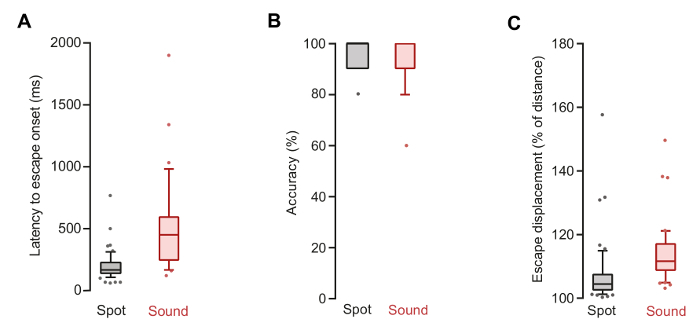

**Figure 2: Escape responses are fast and accurate goal-directed behaviors. **(A) Latency to escape onset from the onset of visual or auditory stimulation. (B) Accuracy of navigation to the shelter during escape, measured such that the value is 100% if the mouse runs directly to the shelter, and 10% less for each hole away from the shelter (*e.g*., 80% if the hole reached first was two holes away from the correct one). (C) Displacement of the mouse during escape plotted against the linear distance between the animal's initial position and the target location, showing that flight is close to linear. Box and whiskers show data from 10-90 percentile and the remaining data points are shown as circles. Red color plots are data from auditory stimulation (n = 36 responses from 15 animals) and black are from visual stimulation (n = 51 responses from 26 animals). Please click here to view a larger version of this figure.

**Figure:**
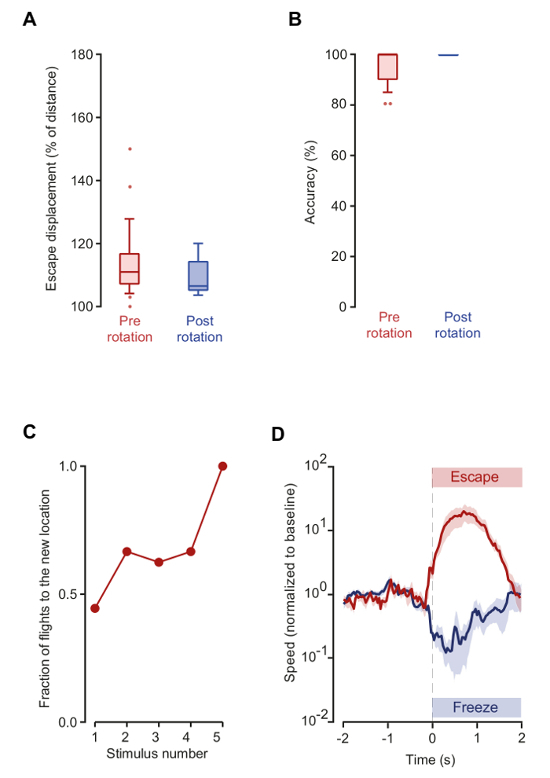

**Figure 3: Mice do not use proximal visual cues or shelter-related cues to guide escape responses. **(A) Displacement plot as in Figure 2C, for the responses pre- and post-rotation of the platform. (B) Accuracy of navigation during escape in pre- and post-rotation conditions. Post-rotation accuracy is measured in reference to the initial location of the shelter, and shows that the mice navigate back to the original shelter location instead the new position. (C) Fraction of escape responses directed towards the new shelter location plotted against stimulation number. After the 5^th^ presentation of the stimulus, all animals escape directly towards the new shelter location (n = 9 animals). (D) Mean speed across the animals in response to visual stimulation with a shelter available (red) or absent (blue), showing escape and freezing responses, respectively. Speed has been normalized to baseline and aligned to reaction time (defined as either the beginning of a flight or freezing response, dashed line). Shaded areas show SEM. (n = 7 animals). For 2A and 2B, the box and whiskers show data from 10-90 percentile and the remaining data points are shown as circles. Red color plots are data from auditory stimulation before platform rotation (n = 25 responses from 8 animals) and blue are from responses after rotating the platform (n = 8 responses from 8 animals). Please click here to view a larger version of this figure.

## Discussion

The assays we describe here are technically straightforward to perform, and with the exception of the platform rotation experiments, can be readily implemented in a standard Barnes maze. Nevertheless, a few complications may arise: on the one hand, the mouse may seem afraid of entering the shelter, which may be due to the shelter not being sufficiently cleaned or dried. On the other hand, the mouse may stay inside the shelter for very long periods of time. It is critical for these experiments that animals are never removed from the shelter manually, as this can disrupt path integration as well as distress the animals and change their response to threat. We suggest terminating the experiment after 90 min and retesting after 48 h if necessary. Another relevant practical consideration is that when the mouse investigates the edge of the platform, it may not detect visual stimuli if its head is angled over the edge, and thus we recommend withholding the stimuli in such situations. Additionally, mice may jump down off the platform on rare occasions. We suggest ending the experiment and retesting after 48 h. Mice that actively leave the platform once are likely to do it again in the future and may have to be excluded from the study. Finally, from our experience, most mice will respond to the visual stimulus (34/36 mice). However, if the mice do not show any type of response to the visual stimulus (either a startle, a flight or a freezing response), they should be excluded from the study.

An important point to consider is that the control of sensory cues is typically a problem in spatial memory assays, as often it is difficult to eliminate all possible sources of contaminating cues. Our experimental setup reduces external visual cues by surrounding the maze with a wall, and it is placed inside a sound-dampening cabinet for sound isolation. To minimize olfactory cues, we recommend thoroughly cleaning the set-up with 70% ethanol or acetic acid between experiments.

Our behavioral assay adds to previous methods for studying spatial navigation^26^ by using instinctive defensive behaviors to probe spatial memory and focusing on ethological scenarios. A critical difference between the procedures described here and the standard BM assay is the lack of training sessions. In our assays, the habituation period ensures that the mouse visits the shelter at least once and often many more times, which we have previously shown to be sufficient to memorize the shelter location^2^. We believe that an important reason for the high success rate and accuracy in finding the shelter despite the lack of training is never passively displacing the animal during the experiment, thus making path integration a viable navigation strategy. We note however that our assay deals with memory formed and assessed during a single session, and that we have not tested for long-term memory of shelter location, which is usually the objective of standard BM experiments. Finally, by using discrete innately aversive stimuli rather than the commonly used fan or buzz for the duration of the session, our assay provides very good experimental control over two behaviors that might use different navigation strategies: foraging and shelter-directed escape. We believe that the use of these assays in combination with modern neuroscience techniques for recording and manipulating neural activity can provide important insights into how neural circuits compute behavior.

## Disclosures

Most of the data included in the results section is a subset of the data presented in Vale *et. al* 2017.
